# The Challenge of Treatment in Potential Celiac Disease

**DOI:** 10.1155/2019/8974751

**Published:** 2019-10-20

**Authors:** Chiara Maria Trovato, Monica Montuori, Francesco Valitutti, Beatrice Leter, Salvatore Cucchiara, Salvatore Oliva

**Affiliations:** ^1^Pediatric Gastroenterology and Liver Unit, Department of Pediatrics, Sapienza University of Rome, Rome, Italy; ^2^European Biomedical Research Institute of Salerno, Salerno, Italy; ^3^Department of Molecular Medicine, Sapienza University of Rome, Rome, Italy

## Abstract

Potential celiac disease (PCD) is defined by the presence of positive serum antibodies, HLA-DQ2/DQ8 haplotypes, and a normal small intestinal mucosa (Marsh grade 0-1). This condition occurs in one-fifth of celiac disease (CD) patients and usually represents a clinical challenge. We reviewed genetic, histologic, and clinical features of this specific condition by performing a systematic search on MEDLINE, Embase, and Scholar database. Accordingly, we identified different genetic features in patients with PCD compared to the classical forms. Frequently, signs of inflammation (deposits of immunoglobulin A (IgA) and/or increased number of intraepithelial lymphocytes) can be clearly identify in the mucosa of PCD patients after an accurate histological assessment. Finally, the main challenge is represented by the treatment: the gluten-free diet should be considered only in the presence of gluten-dependent symptoms in both children and adults. *What is known*: (i) potential celiac disease (PCD) occurs in one-fifth of all celiac diseases (CD), and (ii) despite the absence of classical lesions, clear signs of inflammation are often detectable. *What is new*: (i) patients with PCD show different genetic features, and (ii) the presence of gluten-dependent symptoms is the main determinant to initiate the gluten-free diet, after a complete diagnostic work-up.

## 1. Potential Celiac Disease

Celiac disease (CD) is a systemic disorder caused by gluten and characterized by the presence of a variable combination of gluten-dependent clinical manifestations, CD-specific antibodies, HLA-DQ2 or HLA-DQ8 haplotypes, and enteropathy [1]. Potential CD (PCD) is the condition related to people with a normal (Marsh grade 0) or minimally abnormal (Marsh grade 1) intestinal mucosa who are at increased risk of developing CD, as indicated by both positive serum endomysial (EmA) and tissue transglutaminase antibodies (tTGA2) and a positive histocompatibility leukocyte antigen (HLA-DQ2 or HLA-DQ8) genotype [2]. Symptoms and signs of the disease are not always clinically manifest, and even when present, they can range from mild to severe.

The term “potential CD” was first introduced by Ferguson in 1993 [3], and it has long been used interchangeably with “latent CD”; however, the latter has recently been discontinued, as suggested by the Oslo definition [2]. The diagnosis of PCD has significantly increased in the last years as a result of increased CD screening in the general population [4–6]. The number of patients with PCD is now sizeable, and this condition represents about one-fifth of total CD patients [7]. Compared with active classical CD, PCD is characterized by features including lower prevalence of DQ2 and higher prevalence of DQ8 [8]. Patients with PCD more frequently show low-to-moderate HLA-related risk; these cases bear half of the DQ2 heterodimer, either DQB1^∗^02 or DQA1^∗^05 only. Furthermore, six polymorphisms have been differently distributed in potential CD; these factors could be implicated with CD pathogenesis maybe with a “gene-dosage” effect as reported for HLA [9]. Establishing a certain diagnosis of PCD is of the utmost importance. False positive values of antibodies can be determined by analytical or random errors in the assay. Conversely, negative histological findings can be generated by a small number of biopsies due to “patchy” involvement of the bulb and duodenal mucosa [10–13], inappropriate biopsy orientation, the lack of the pathologists' expertise [14, 15], and an inadequate gluten intake before the endoscopy [16].

## 2. Histology Features and Prognostic Biomarkers

**Figure 1:**
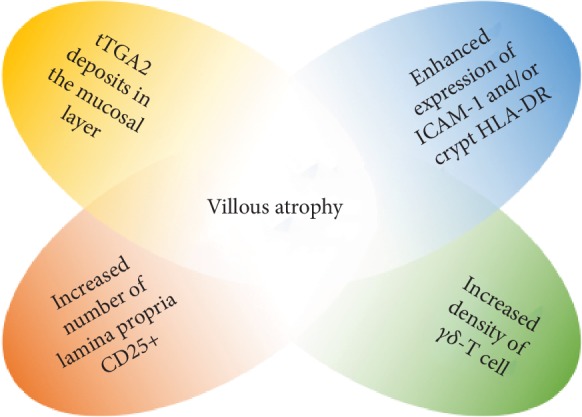
The four most important prognostic factors for villous atrophy in PCD.

In PCD, despite the absence of severe mucosal damages, clear signs of inflammation are often present. There is a remarkable research activity to improve the diagnosis and identify initial mucosal changes in PCD: the four most important prognostic factors for villous atrophy are described in Figure 1. A short history of the most important findings concerning PCD is reported in Table 1, and results from these studies are here described more in detail.

Paparo et al., in 2005, showed immunohistochemical features of immune activation in the epithelium, lamina propria, and crypts in PCD: 70.8% of PCD patients presented an increased number of lamina propria CD25+ and/or enhanced expression of ICAM-1 and crypt HLA-DR [17]. It has been hypothesized that circulating antitissue transglutaminase 2 (tTGA2) may be the result of a “spillover” from the intestinal mucosal layer [18, 19]. Therefore, identifying anti-tTGA2 deposits in the mucosal layer can be a key factor in the histological assessment of CD: such deposits have been reported below the epithelial layer and around blood vessels in both pediatric and adult patients with overt CD [20, 21]. These features could also have a predictive role for villous atrophy, since they have been described in early-stage CD [22]. In 2006, Salmi et al. demonstrated that the detection of anti-tTGA2 deposits in the mucosa seems to be rather specific for CD and might be helpful in predicting the evolution to more severe histological damage [23]. The same data have been discussed in a recent review and, in the same way, have been considered as “markers of existing early disease” [24].

tTGA2 deposits were observed by Tosco et al. [25] following a patchy distribution with areas of clear positivity and areas with absent signal, as already described in mucosal damage of active CD [10, 13]; however, these deposits can also be found only in bulb duodenal biopsies [26]. In 2017, an Italian study demonstrated that in at-risk infants for CD, detection of mucosal deposits of anti-tTG2 IgA resulted in 88.3% positive predictive value [22]. The prevalence of *γδ* T-cell has also been suggested as a histological biomarker of CD. In fact, an increase in intraepithelial lymphocytes at the villus tip and a high *γδ*+ intraepithelial cell count can be considered good predictors of CD in patient with PCD, as described by two different studies from Finland [27, 28]. Some authors suggest that high density of *γδ* T-cell receptor-bearing intraepithelial lymphocytes (IELs) can be a prerequisite for developing CD in patients with no morphological abnormality, yet carrying the susceptibility genes; however, despite an increased density of *γδ* T-cell in potential CD, these findings cannot be considered pathognomonic for celiac disease [29, 30]. It has been hypothesized that in PCD, the intestinal mucosa is maintained architecturally normal by an increased enterocyte proliferation, which will end up in a reduced enterocyte maturity and will thus lead to reduced absorptive capacity of the small bowel [31]. In the same year, another study demonstrated how T-cells seem to be activated and differentiating toward a Th1 pattern, as suggested by high levels of interleukin-2 (IL-2), interferon-*γ* (IFN-*γ*), and TGF-*β* transcription factor. The same study showed an increased density of CD4+CD25+Foxp3+ T regulatory cells, which exert suppressive effect not impaired by IL-15 in potential CD [32]. A recent paper from Borrelli et al. in PCD patients showed reduced expression and increased upregulation in the presence of specific stimuli of interleukin-21 (IL-21), an important cytokine regulating innate and adaptive immune response, differently from active CD. In this study, PCD density of IL-21-producing cells in the lamina propria was found to correlate with serum titer of tTGA2, suggesting a lack of ability of IL-21 to enhance and maintain chronic inflammation in early phases of disease in active or potential CD [33]. In active CD, the overexpression of IL-21 is likely to play a crucial role in the activation of cytotoxic T-cells leading to epithelial cell death and mucosal destruction [34]. Aside from immunological controversies, an overlapping metabolomic signature was found for PCD and active disease, suggesting that common functional-biochemical stigmata might call for the same dietary treatment [35].

## 3. To Treat or Not to Treat?

The therapeutic management of PCD patients represents the main challenge. The only accepted treatment for CD is gluten-free diet (GFD), but the treatment for potential celiac disease still remains unclear. Likewise, there is no clear consensus in the PCD follow-up [36]. The natural history of PCD, both in adults [7] and children [25], is not sufficient to recommend GFD in any patient. Recently, Auricchio et al. [37] developed a model to predict the evolution to villous atrophy in PCD. They suggested GFD when symptoms of CD can be clearly detected, even without a mucosal damage. This approach aims at reducing symptoms and antibody titers (tTGA2 and EMA), as well as healing minimal alterations in intestinal mucosa [1]. Conversely, the use of GFD in asymptomatic patients is still debated. In 2009 and 2010, two studies from Finland showed that both adults [38] and children [39] with PCD obtained a clinical response to GFD regardless of the presence of small-bowel lesions. According to these studies, the authors suggested to start the dietary treatment as early as possible since treatment would result in reduced risks of delayed puberty and gynecological issues, while avoiding effects on bone mineralization, dental enamel development, and growth. Conversely, in a recent review, Itzlinger et al. considered GFD as inappropriate treatment in asymptomatic patients with PCD [40].

Diverging results emerged from Mandile's work, in which only 54% of PCD symptomatic patients have a positive clinical response during the first 12 months of GFD. However, the authors speculated about irritable bowel syndrome as a significant confounding factor in these patients [41]. In 2014, Auricchio et al. demonstrated that a considerable proportion of PCD patients usually had a fluctuation or decrease of antibody levels, while in those with persistently positive anti-TG2 under a free diet, the mucosal damage was not detectable in 66% of cases until 9 years of follow-up [42]. In 2019, Lionetti et al. reached similar conclusions: in PCD children on free diet, the risk of progression to overt CD is trivial [43].

Previously, Tosco et al. demonstrated that approximately 33% of asymptomatic children with PCD would develop villous atrophy after 3 years without prescribing a GFD [25]. The authors suggested that most children with potential celiac disease remain healthy and for these reason only symptomatic children would start GFD.

**Figure 2:**
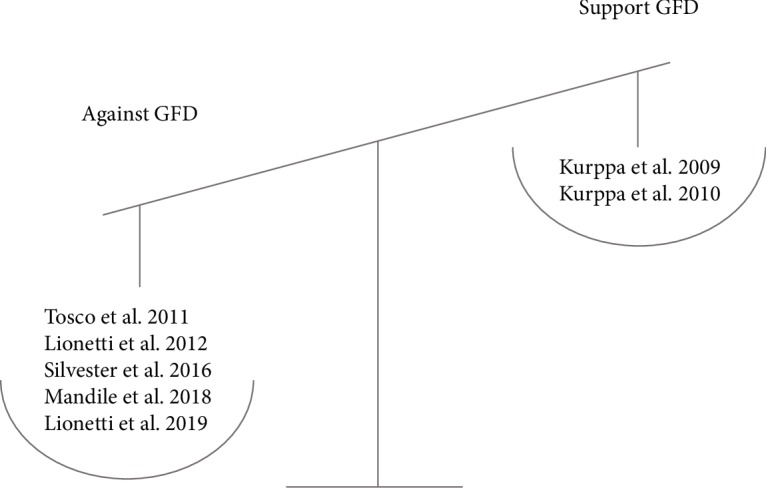
Results of available evidence in support or against GFD in PCD asymptomatic patients.

In 2012, a decision tree for asymptomatic children with tTGA values lower than 11-fold the upper limit normal was proposed [44]. Symptomless children with a family history of CD and positive CD markers could initially remain on normal free diet, particularly in the case of modest tTGA titer increase. Biopsies should be recommended after a persistent antibody positivity for at least 3-6 months. In 2016, another group indicated that asymptomatic patients can be monitored for the development of new symptoms and/or substantial increase in serum tTGA2 antibodies [45]. These studies are summarized in Table 2 and Figure 2.

**Figure 3:**
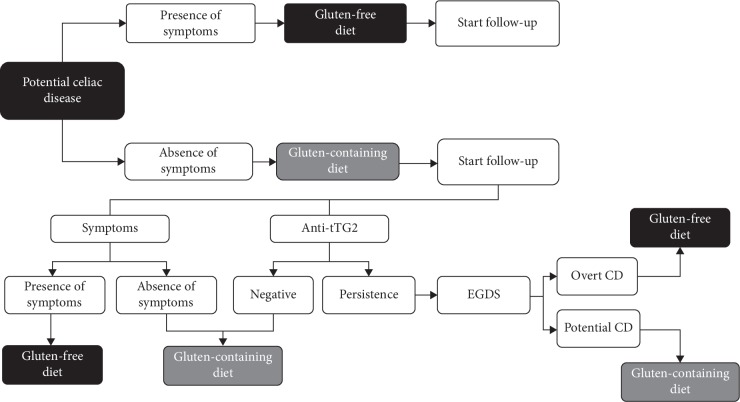
Diagnostic algorithm for PCD.

In conclusion, the presence of symptoms in both adults and children should be considered as the main determinant to prescribe a GFD in potential celiac disease. It is important to remember that all symptoms have to be considered important for the beginning of a GFD. There is no difference in the decision tree, in fact, if patient has gastrointestinal (diarrhea, constipation, abdominal pain) or extraintestinal manifestation (anemia, osteoporosis, migraine), as suggested by Popp and Maki in a recent review too [24]. As the timing of flattening is totally unpredictable, asymptomatic patients with PCD should undergo a comprehensive follow-up in order to detect early symptoms and promptly start a GFD. A conclusive algorithm is proposed in Figure 3 with the aim to provide valuable information in the management of this challenging condition.

Further research is necessary in order to establish the optimal frequency of testing the antibodies and clinical evaluation for PCD patients (both adults and children) continuing after initial evaluations on gluten-containing diet. Dietary habits and gluten intake during clinical evaluation should be routinely checked during clinical evaluation, as following a diagnosis of PCD, the patient or his family could decrease the amount of gluten, resulting in false negative serology and fluctuating antibodies.

## Figures and Tables

**Table 1:** A short history of the most important findings concerning PCD.

Study	Year	Conclusions
Holm et al. [29]	1992	A healthy person who initially has a normal biopsy, but who also has an increased density of *γδ* T-cells, may later develop mucosal atrophy compatible with CD.
Iltanen et al. [30]	1999	39 of 79 (49%) children with normal jejunal mucosa had an increased density of intraepithelial *γδ* T-cells.
Jarvinen et al. [28]	2003	An increase especially in *γδ* T-cells strengthens the probability of CD.
Korponay-Szabo et al. [20]	2004	TG2-related IgA deposits in the morphologically normal jejunum were predictive of forthcoming overt coeliac disease with villous atrophy.
Jarvinen et al. [27]	2004	The villous tip intraepithelial lymphocyte count was statistically significantly higher in patients with early-stage coeliac disease than in nonceliac controls (sensitivity, 0.84; specificity, 0.88).
Paparo et al. [17]	2005	Increased number of lamina CD25+ and/or enhanced expression of ICAM 1 and crypt HLA DR.
Salmi et al. [23]	2006	Intestinal coeliac autoantibody deposit had a sensitivity and specificity of 93% and 93%, respectively, in detecting subsequent coeliac disease.
Koskinen et al. [21]	2010	Mucosal transglutaminase 2-specific autoantibody deposits proved to be accurate gluten-dependent markers of celiac disease.
Tosco et al. [25]	2011	In most positive cases a patchy distribution of the deposits was observed with areas of clear positivity and areas with absent signal.
Bernini et al. [35]	2011	Potential CD largely shares the metabolomic signature of overt CD. Results prove that metabolic alterations may precede the development of small intestinal villous atrophy.
Biagi et al. [31]	2013	In PCD, the intestinal mucosa is maintained architecturally normal thanks to an increased enterocytic proliferation.
Borrelli et al. [32]	2013	Potential CD patients show a low grade of inflammation that could likely be due to active regulatory mechanism preventing the progression toward a mucosal damage.
Borrelli et al. [33]	2016	In potential CD, IL-21 is less expressed than that in active CD.
Borrelli et al. [22]	2018	In CD, the intestinal deposits of anti-tTG2 are a constant presence and appear very early in the natural history of the disease.

**Table 2:** Results of available evidence in support or against GFD in PCD asymptomatic patients.

Study	About GFD	Study population	Conclusions	Limitations
Tosco et al. [25]	Against GFD	106 children	33% of incidence of villous atrophy after 3 years in with PCD	Unknown number of patients lost at follow-up
Lionetti et al. [44]	Against GFD	24 asymptomatic children	CD markers disappear in most young children with potential CD despite a regular diet	Small sample size
Silvester et al. [45]	Against GFD	*Review paper*	In the absence of symptoms or villous atrophy, treatment with a GFD does not appear to be necessary in most cases	N/A
Mandile et al. [41]	Against GFD	47 children	Association between CD and irritable bowel syndrome may be a significant confounding factor	Irritable bowel syndrome is overlapping with CD
Lionetti et al. [43]	Against GFD	23 asymptomatic children	Risk of progression to overt CD while on a gluten-containing diet is very low in the long-term.	Age of the study group and study design
Kurppa et al. [38]	Supports GFD	23 adults	Patients with endomysial antibodies benefit from a GFD regardless of the degree of enteropathy.	Marsh II included in study population
Kurppa et al. [39]	Supports GFD	17 children	Children benefit from early treatment despite normal mucosal structure	Small sample size
